# Comparison of Effects of Plasma Surface Modifications of Bamboo and Hemp Fibers on Mechanical Properties of Fiber-Reinforced Epoxy Composites

**DOI:** 10.3390/polym16233394

**Published:** 2024-11-30

**Authors:** Pornchai Rachtanapun, Choncharoen Sawangrat, Thidarat Kanthiya, Kannikar Kaewpai, Parichat Thipchai, Nuttapol Tanadchangsaeng, Patnarin Worajittiphon, Jonghwan Suhr, Pitiwat Wattanachai, Kittisak Jantanasakulwong

**Affiliations:** 1 Faculty of Agro-Industry, Chiang Mai University, Chiang Mai 50100, Thailand; pornchai.r@cmu.ac.th; 2 Center of Excellence in Materials Science and Technology, Chiang Mai University, Chiang Mai 50200, Thailand; patnarin.w@cmu.ac.th; 3 Center of Excellence in Agro Bio-Circular-Green Industry, Faculty of Agro-Industry, Chiang Mai University, Chiang Mai 50100, Thailand; 4 Department of Industrial Engineering, Faculty of Engineering, Chiang Mai University, Chiang Mai 50200, Thailand; choncharoen@step.cmu.ac.th; 5 Office of Research Administration, Chiang Mai University, Chiang Mai 50200, Thailand; thidaratkanthiya05@gmail.com; 6 Science and Technology Park (STeP), Chiang Mai University, Chiang Mai 50100, Thailand; kannikar@step.cmu.ac.th; 7 Nanoscience and Nanotechnology, Faculty of Science, Chiang Mai University, Chiang Mai 50200, Thailand; parichat_thi@cmu.ac.th; 8 College of Biomedical Engineering, Rangsit University, Pathumthani 12000, Thailand; nuttapol.t@rsu.ac.th; 9 Department of Chemistry, Faculty of Science, Chiang Mai University, Chiang Mai 50200, Thailand; 10 School of Mechanical Engineering, Sungkyunkwan University, Suwon-si 16419, Republic of Korea; suhr@skku.edu; 11 Department of Civil Engineering, Faculty of Engineering, Chiang Mai University, Chiang Mai 50200, Thailand

**Keywords:** plasma, surface modification, bamboo, hemp, epoxy composites

## Abstract

In this study, we investigated the behaviors of epoxy composites reinforced with bamboo (BF) and hemp (HF) fibers. Both fibers were treated using dielectric barrier discharge (DBD) plasma for various durations (2.5 to 20 min). Epoxy resin (ER) was mixed with BF or HF with and without plasma treatment. The Fourier-transform infrared spectra of the plasma-treated fibers showed an enhanced peak intensity of carboxyl groups. ER/BF treated for 20 min exhibited a high tensile strength (up to 56.5 MPa), while ER/HF treated for 20 min exhibited a more significant increase in elongation at break (6.4%). Flexural tests indicated that the plasma treatment significantly improved the flexural strength of the hemp composites (up to 62.2 MPa) compared to the bamboo composites. The plasma treatment increased the fiber surface roughness and interfacial bonding in both composites. The thermal stability and wettability were improved by the DBD plasma treatment. The DBD plasma treatment enhanced the interfacial adhesion between fibers and ER matrix, which improved the mechanical, thermal, and wettability properties of the composites.

## 1. Introduction

In recent years, biocomposites have emerged as versatile materials in various industries [1,2]. They are environmentally friendly material alternatives for the replacement of petroleum-based polymers due to their ability to combine the strengths of polymers with reinforcing materials. The reinforced polymers by biomaterials exhibit enhanced mechanical, thermal, and chemical properties. Composites are produced by embedding a reinforcing phase within a polymer matrix [3], which provides lightweight structures, strength, and durability. The use of cellulose fibers, such as bamboo, flax, sisal, jute, and kenaf, in polymer composites as reinforcement gained popularity in several engineering applications due to the low cost, density, favorable mechanical properties, and recyclability [4,5]. Cellulose fibers are environmentally friendly, nontoxic, and renewable materials. Manufacturing industries, such as the packaging, building construction, automotive, and furniture industries, have been encouraged to use plant fibers instead of reinforcing materials [6,7,8].

Natural fibers are a biomaterial used to reinforce a polymer matrix. Bamboo belongs to the grass family poaceae, which is known for its strength and ability to thrive in diverse climates [9]. Bamboo is composed of a series of nodes and internodes, which yields a hollow cylindrical structure. The cellulose and lignin contents of bamboo fibers are up to 40–50% and 20–30%, respectively, which provides rigidity, high tensile strength, and high stiffness [10,11]. This composition makes bamboo a strong and flexible material, which can be used for various construction and industrial applications. In the context of fiber reinforcement, bamboo is processed into bamboo fibers (BFs) for use in polymer composites. These fibers exhibit a high tensile strength, comparable to those of some synthetic fibers [12,13]. Bamboo fiber composites are increasingly used in construction, automotive components, and packaging due to their lightweight structure, strength, and biodegradability. Hemp is another natural alternative for composite reinforcement. Hemp fibers (HFs) are strong and lightweight, and exhibit favorable thermal properties, making them ideal for composites requiring enhanced toughness and thermal stability. HF contains cellulose (60–70%), hemicellulose (15–25%), and lignin (5–10%), which provides excellent mechanical properties. Hemp has been used in textiles for centuries. Its potential as a reinforcing fiber in polymer composites is particularly recognized in sustainable manufacturing sectors [14,15]. However, the hydrophilic property of plant fibers, hydrophobic property of resins, and compatibility problems limit the application of composites [16]. Surface treatments with chemical and physical methods are employed to enhance the interfacial adhesion between natural fibers and matrix resins. Substantial studies have been carried out to improve interfacial adhesion by chemical, physical, or other modification methods. Common surface modification methods include alkali, silane, plasma, and enzymatic treatments [17,18,19,20,21]. These treatments improve the fiber surface by increasing the roughness or introducing functional groups that enhance the adhesion, which leads to transfer load between the fiber and matrix [22,23]. Natural fiber composites are increasingly used in construction, automotive components, and packaging [24,25] due to their lightweight structure, strength, and biodegradability.

Plasma treatment involves exposure of a material surface to a partially ionized gas (plasma), electrons, ions, and neutral particles. The interaction between the plasma and surface induces chemical, physical, and structural modifications of materials [26]. The use of dielectric barrier discharge (DBD) plasma is a specific form of atmospheric plasma treatment with a dielectric barrier that separates two electrodes. This setup provides uniformity of nonthermal plasma generation at atmospheric pressure. The DBD plasma is frequently used to treat natural fibers due to its energy efficiency and scalability. In the DBD plasma, various gasses can be used, such as O_2_, N_2_, or Ar, depending on the desired surface modification [27,28,29]. Natural fiber surface improvement by DBD plasma is a novel process to increase roughness and reactive functional groups on fiber surface to form physical and chemical crosslinking with the polymer matrix, respectively. The synergistic crosslinking power may increase properties of polymer composite which can be applied for construction, electrical, coating, packaging, agriculture, and medical applications.

Epoxy resin (ER) is the most used thermoset structure in engineering fields owing to its chemical resistance, excellent mechanical properties, and electrical insulation [30]. ERs are created through reactions between epoxides (three-membered cyclic ethers) and curing agents (hardener agents), such as amines, anhydrides, or phenols. Upon curing, epoxy forms a rigid crosslinked network structure that provides the characteristic strength and durability [31]. Fiber-reinforced epoxy composite is a material obtained by embedding high-strength fibers into an ER matrix. This combination creates a composite material with superior mechanical properties compared to those of the individual components. The fibers act as a reinforcing material to provide strength and stiffness, while the ER acts as a matrix that binds the fibers together, distributes stress, and protects the fibers from damage [32,33]. Some researchers attempted to develop natural fiber-reinforced ERs without surface modification, which led to weak mechanical properties and connecting phase between fibers and the ER matrix. The use of chemical and physical processes is a target approach to improve the fiber surface before blending with ER. When the surface of the fiber is improved by chemical and physical roughness, this improves the reaction, interfacial adhesion, and properties of the blend.

Therefore, in this study, a combined surface modification, with chemical and physical processes, by the plasma technology was used to improve the surface of BF and HF. BF and HF were plasma-treated using an argon + oxygen gas (Ar + O_2_) for different times of 0 to 20 min, followed by plating in an NH_4_OH solution to improve the chemical structure of the fibers. NH_4_OH was used to provide grafting −NH_2_ groups onto the surface structure of the fibers, which can react with epoxy groups of ER. The DBD plasma technique was used to improve the roughness surface and polarity of the fibers. The properties of both fiber composites were investigated. Fourier-transform infrared (FTIR) spectroscopy was utilized to evaluate the chemical bonding and reaction mechanisms within the composites, while tensile strength and flexural properties were evaluated to assess the impact of the plasma treatment. Morphological and thermal stability studies were carried out to explain the impact of the plasma treatment on the composite structure and performance. This research provides insights into the potential of plasma-treated natural fiber composites in creating stronger and more durable ecofriendly materials for industrial applications.

## 2. Materials and Methods

### 2.1. Materials

BFs were obtained from South Samoeng, Chiang Mai, Thailand. HFs were purchased from Royal Project Foundation, Chiang Mai, Thailand. ER was of grade A302. A hardener of grade A301 was purchased from Easy Resin, Co., Ltd., Nonthaburi, Thailand. All chemicals for the surface modification, including sodium hydroxide (NaOH), sodium chlorite (NaClO_2_), and ammonium hydroxide (NH_4_OH), were purchased from Merck & Co. Inc., Darmstadt, Germany.

### 2.2. Surface Treatment of Fibers

**Figure 1:**
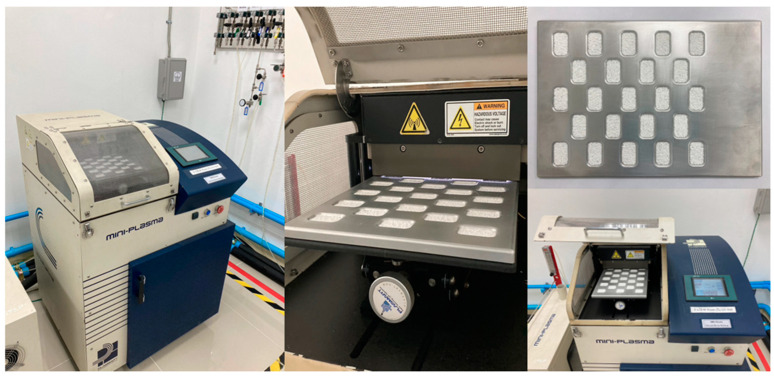
Dielectric barrier discharge (DBD) plasma machine.

Before the plasma treatment, bamboo and hemp fibers were mildly alkaline treated in an aqueous NaOH solution (20% *w*/*v*) at 80 °C for 5 h. The pulps were bleached with NaClO_2_ to remove lignin and hemicellulose, as described in our previous report [28]. The fibers were sieved through a 180 µm sieve. The bamboo and hemp fibers were treated for surface modification using a DBD plasma approach. The DBD plasma machine is shown in Figure 1. The fibers were put on the tray, while DBD was generated through two parallel high-power electrodes covered by a thin quartz foil. The grounded electrode can be adjusted to change the discharge gap for the fibers. The DBD plasma was generated by a high-voltage power of 180 W (3.45 W/cm^2^) with a constant frequency of 13.56 kHz. Ar + O_2_ gasses were used for plasma flow rates of 8 and 10 L/min, respectively. The DBD plasma was applied on the bamboo and hemp fibers for 2.5, 5, 10, 15, and 20 min. The treated fibers under each plasma condition were modified on the surface by mixing into an NH_4_OH solution (1:10% *w*/*v*). The sample was heated by a hot plate at 60 °C and stirred continuously for 1 h. Afterward, the NH_4_OH solution was evaporated from the final product by heating at 60 ± 2 °C for 48 h.

### 2.3. Fiber Composite Material Fabrication

The fiber composite materials were fabricated by the hand layup technique followed by the mixing of ER and hardener (2:1% *w*/*w*), as indicated in Table 1. The fiber composites were separated into untreated (F_untr_) and treated (F_tr_) plasma groups for the different natural fiber types (bamboo and hemp), and then the fiber samples were mixed with ER at room temperature. The mixed samples were stirred to enhance the dispersion of fibers, while air bubbles were removed in the ER with an aspirated vacuum. The mixture was then cast into a silicone mold of a bone shape followed by drying at 80 °C for 5 h.

### 2.4. FTIR Spectroscopy

The chemical functional groups of the samples were examined using FTIR spectroscopy (Thermo Nicolet 6700 FTIR spectrometer, Thermo Fisher Scientific, Woodland, CA, USA) with the ATR mode. FTIR spectra were acquired in the range of 500–4000 cm^−1^ (32 scans, scan resolution of 4 cm^−1^).

### 2.5. Tensile Properties

The bone-shaped configuration samples were prepared with dimensions of 2 mm × 5 mm × 1 mm (width × length × thickness). The test was carried out according to the JISK-6251-7 standard (Model MCT1150, Tokyo, Japan) at room temperature. The elongation at break (EB) and tensile strength (TS) were measured at a crosshead speed of 50 mm/min. Ten replicates were performed on each sample.

### 2.6. Flexural Test

The samples were cast into the silicone mold with dimensions of 13 mm × 65 mm × 3 mm (width × length × thickness) according to the American Society for Testing and Materials (ASTM) D790 standard. The testing was conducted using a universal testing machine (H1KS, Hounsfield Test Co., Ltd., Surrey, UK) in a three-point bending configuration with a force of 1 kN at room temperature. The average from five samples was recorded for the analysis. The flexural strength and modulus were calculated by the following [28]:

(1) $$\mathrm{Flexural}\;\mathrm{strength}=\frac{3\mathrm{FL}}{2bd^{2}},$$

(2) $$\mathrm{Flexural}\;\mathrm{modulus}=\frac{mL^{3}}{4bd^{3}}$$

where *F*, *L*, *b*, *d*, and *m* are the maximum failure load (N), length of span (mm), width, thickness, and slope of the load–displacement curve tangent to the initial line, respectively.

### 2.7. Morphological Analysis

The fractured morphologies of the samples were studied by scanning electron microscopy (SEM; JSM-IT300LV, JEOL Co., Ltd., Tokyo, Japan). The impact fracture surface broken in liquid nitrogen was evaporated in a hot air oven at 60 °C for 2 h. The samples were sputter-coated with gold and observed at 15 kV under a vacuum.

### 2.8. Thermal Stability

The thermal stabilities of the epoxy and fiber-reinforced epoxy composites were evaluated by a thermogravimetric analysis (TGA; Mettler Toledo STARe TGA/DSC3+, Greifensee, Switzerland) under a nitrogen atmosphere. The testing temperature range was 25–600 °C, while the rate was 10 °C/min.

### 2.9. Contact Angle

The water contact angle was measured using a water droplet instrument (DSA30E, Krüss Co., Ltd., Hamburg, Germany). Samples were formed by casting them into a silicone mold and pasting them on glass slides. The water drop shape was recorded at 0–10 min. At least five measurements of each sample were performed to average the water contact angles.

### 2.10. Statistical Analysis

A statistical comparison was carried out using a one-way analysis of variance (ANOVA) with the SPSS software (SPSS version 17, Armonk, NY, USA). The statistical significance (*p* < 0.05) was estimated using the Duncan test.

## 3. Results and Discussion

### 3.1. FTIR Spectroscopy

**Figure 2:**
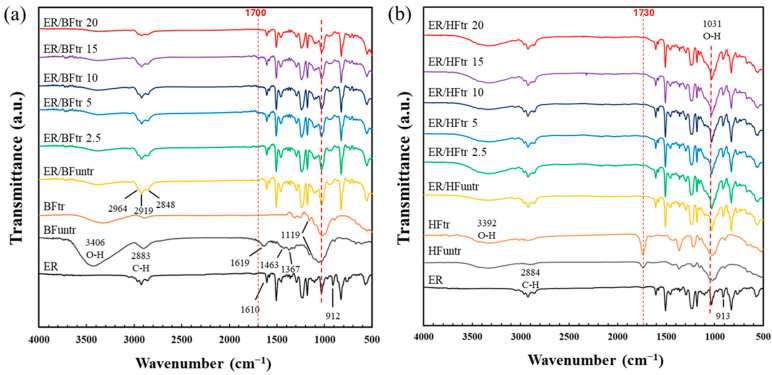
FTIR spectra of (**a**) bamboo and (**b**) hemp fiber-reinforced epoxy composites (untreated and treated with Ar + O_2_ for 2.5–20 min + NH_4_OH).

The chemical characteristics of the fiber-reinforced epoxy composites evaluated by FTIR spectroscopy for bamboo and hemp fibers with DBD plasma treatment are shown in Figure 2. The untreated and treated fibers exhibited hydroxyl (O−H) stretching vibrations at 3330 to 3400 cm^−1^ [34,35]. The band at 2890 cm^−1^ was assigned to the C−H stretching vibration of the cellulose fiber containing a functional group of alkanes [36], while the band at 1635 cm^−1^ was attributed to the aromatic of lignin [37,38]. The peak around 1730 cm^−1^ was indicated to the carbonyl (C=O) stretching of acetyl groups of hemicellulose [39]. Both plasma-treated and untreated fibers exhibited peak disappearance of lignin at 1635 cm^−1^ partly due to the alkaline treatment and blenching before the plasma treatment. In the ER/BF and ER/HF composites, ER exhibited C−C stretching peaks at 1610 and 1585 cm^−1^ and C=C stretching vibrations of the ER aromatic ring at 1508 and 1454 cm^−1^ [40]. The bands at 1241, 913, and 827 cm^−1^ were C−O symmetrical stretching, C−O asymmetrical stretching, and C−O−C stretching in the oxirane ring, respectively [41,42]. For the plasma-treated composites, an increase in peak intensity at 3200−3600 cm^−1^ was observed due to O−H stretching of hydroxyl groups of the fibers. The treatment with the Ar + O_2_ gas generated the polar groups of carbonyls (C=O) and carboxyl (−COOH) at 1680−1720 cm^−1^. In addition, carbon hydrogen group (CH_2_, CH_3_) stretching was observed at 2850–3000 cm^−1^ [28,43]. The amount of oxygen-containing groups on the surface increased with the time of plasma treatment owing to bonding between the fiber and resin [44]. However, the epoxy groups of ER (912 cm^−1^) were not observed on the ER/BF_tr_ composite (Figure 2a). The ER/BF_tr_ composites exhibited a new shoulder peak at 1700 cm^−1^, which disappeared upon the treatment for 20 min. This new peak indicates a new C−O formation from the reaction between the ER and BF surface, which changed the form with the 20 min treatment. HF_untr_ exhibited –COOH at 1730 cm^−1^ and C−O at 1360 cm^−1^. The intensities of these two peaks increased with the plasma treatment due to the increasing contents of the −COOH and C−O groups on the surface of HF. The results indicate that the −COOH groups react with NH_4_OH and epoxy groups of ER. −COOH groups disappeared after mixing with ER. The results also indicate that −NH_2_ groups graft on the fiber surface and react with epoxy groups of ER. For the ER/HF composites, the intensity of epoxy groups of ER at 912 cm^−1^ decreased with the time of plasma treatment owing to the high reaction rate between the surface of HF and ER. The plasma treatment improves the reactive functional groups of the fibers surface, which react with epoxy groups of the ER main matrix [10,42,45]. The plasma treatment caused changes in the chemical and physical structures of the bamboo and hemp fibers [27,46,47], resulting in the formation of functional groups that induced bonds and interfacial adhesion between EB matrix and the fiber. These reactions could improve the properties of composites.

### 3.2. Tensile Properties

**Figure 3:**
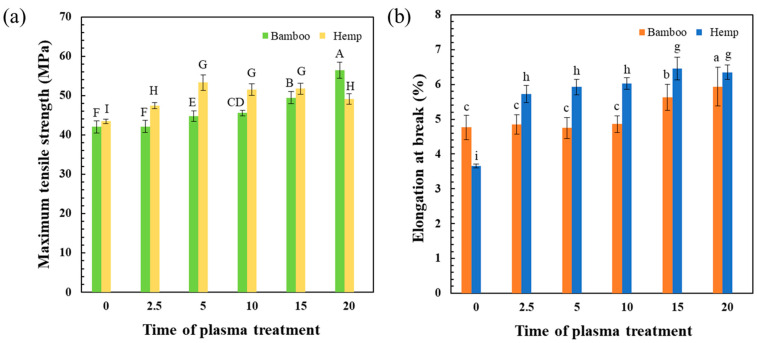
Comparison of tensile properties between bamboo and hemp fiber-reinforced epoxy composites, untreated (0) and treated with Ar + O_2_ for 2.5–20 min + NH_4_OH. (**a**) Maximum tensile strength (MPa) and (**b**) elongation at break (%). The mean values of the tensile strength (uppercase letter) and elongation at break (lowercase letter) differ significantly (*p* < 0.05).

Figure 3 shows the tensile properties of ER composites with plasma treatment (0–20 min) on BF and HF. The tensile strength and elongation at break of the ER/BF_untr_ composite using bamboo untreated fibers were 42 MPa and 4.7%, respectively. The EP/BF_tr_ composites treated with the Ar + O_2_ gas for 2.5–20 min exhibited increasing tensile strength and elongation at break with the treatment time. They exhibited the highest tensile strength at 20 min (56.5 MPa). The tensile strength and elongation at break of the hemp untreated composite (ER/HF_untr_) were 43.4 MPa and 3.6%, respectively. The EP/HF_tr_ composites exhibited higher tensile strengths than that of the untreated composite (highest value of 53.2 MPa at 5 min), which slightly decreased with the increase in treatment time. The elongation at break of the EP/HF_tr_ composite significantly increased with the treatment time from 3.6% to 6.4%. The improved tensile properties of the bamboo and hemp fiber-reinforced epoxy composites were attributed to the adhesion between the fibers and ER matrix. The plasma treatment of the fibers significantly affected the adhesion performance of the inter-face between the reinforced fibers and ER [48]. The samples using the hemp-treated fiber reinforcement were improved in terms of elongation at break, while the bamboo-treated fibers provided a larger improvement in tensile strength. The surface roughness of the fibers increased due to plasma etching, which enhanced the penetration and diffusion at the fiber–matrix interface and generated interlocking bonds [27,49]. The high degree of interfacial chemical reaction between epoxy groups of ER and –COOH groups of fibers with physical crosslinking with a high fiber roughness could improve the mechanical properties of the composites. Optimal plasma processing parameters relate to mechanical properties of the composites [50].

### 3.3. Flexural Test

We evaluated the flexural strengths of the composites according to the plasma treatment of the bamboo and hemp fibers, as shown in Table 2. The flexural strength and modulus of the bamboo untreated composite (ER/BF_untr_) were 55.9 MPa and 7.6 GPa, respectively. The flexural strengths of ER/BF_tr_ treated for 2.5, 5, 10, 15, and 20 min were 54.8, 50.5, 56.8, 58.1, and 57.5 MPa, while the flexural moduli were 6.9, 6.6, 8.2, 8.1, and 7.7 GPa, respectively. The ER/HF_untr_ composite exhibited a flexural strength and modulus of 54.7 MPa and 7.6 GPa, respectively. The flexural strengths (53.9, 47.9, 56.7, 54.4, and 62.2 MPa) and moduli (7.7, 6.7, 7.8, 7.5, and 8.6 GPa) of the hemp fiber-reinforced composites after the plasma treatment exhibited increasing trends with the treatment time due to the improvements in HF_tr_ roughness and interfacial adhesion of the ER/HF_tr_ composites. The DBD plasma treatment with O_2_ gas improved the mechanical properties of both bamboo and hemp [51]. Both flexural stress and modulus of hemp were significantly improved compared to those of bamboo due to the different chemical structure of the hemp fibers with a responsive structural basis to the plasma treatment compared to the bamboo [52,53].

### 3.4. Morphological Analysis

**Figure 4:**
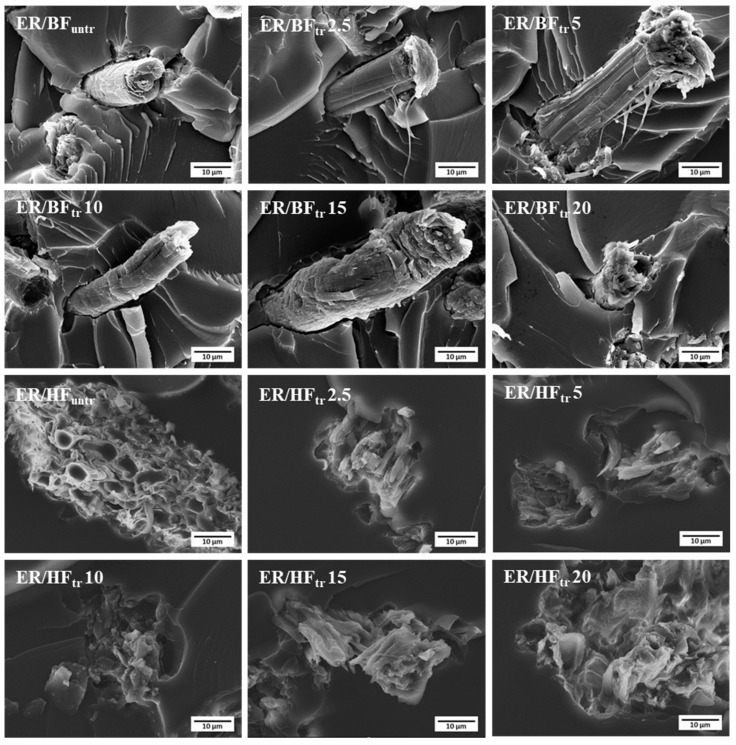
SEM images of BF- and HF-reinforced epoxy composites, untreated and treated with Ar + O_2_ gas for different times at 1000×.

SEM was employed to acquire fractured surface images of the composites. Figure 4 shows the morphologies of the fiber-reinforced epoxy composites with bamboo and hemp structures, untreated and plasma-treated for different times. The ER/BF_untr_ composite with the untreated fibers exhibited a smooth surface of the bamboo fiber similar to that of the ER/BF_tr_ 2.5 sample. The increase in the time of plasma treatment (5–20 min) resulted in a high fiber roughness due to the oxygen in the discharge gasses inducing oxygen groups onto the fiber structure. The plasma treatment induces etching and formation of chemical species on the surface, which corresponds to the roughness [54]. Fine BF_tr_ and HF_tr_ fibers distribution on ER matrix were observed, which provided the properties improvement of composite [55,56]. The bamboo fiber-reinforced composites exhibited obvious gaps between fibers and matrix due to the plasma treatment with O_2_ gas resulting in surface etching and reducing the polarity of the fiber by the formation of oxygen bonding [28,57,58]. Reduction in gaps between the BF surface and ER matrix were observed for the plasma treatment of 20 min, which related to the increased tensile strength. However, ER/HF_tr_20 showed low gaps between HF and ER matrix with reduction in tensile strength due to the occurred degradation of HF fiber which was weaker structure than BF fiber. Combination between interfacial crosslinking and fiber degradation provided slightly decreased tensile strength. The SEM images of the HF-reinforced composites showed different characteristics from those of the bamboo fibers. The morphology of the ER/HF_untr_ composite with untreated fibers clearly exhibited hemp stem sections [59] with characteristics of smooth fibers, large pores, and irregular structure [22,52]. The EP/HF_tr_ composites exhibited low gaps between the HF and BR matrix for all treatment times owing to the high interfacial adhesion between HF and BR via chemical and physical HF surface improvements. The ER/HF_tr_ composites exhibited surface improvements without removed fibers due to the excellent adhesion between the HF and EP [28,60]. The DBD plasma affected the etching of the fiber surface, enhanced the roughness, and induced –COOH groups on the HF structure, due to the surface ion bombardment, etching, and ablation of surface layers [61]. Long-term treatment provided crosslinking reaction which improved interfacial adhesion and mechanical properties of the composites.

### 3.5. Thermal Stability

**Figure 5:**
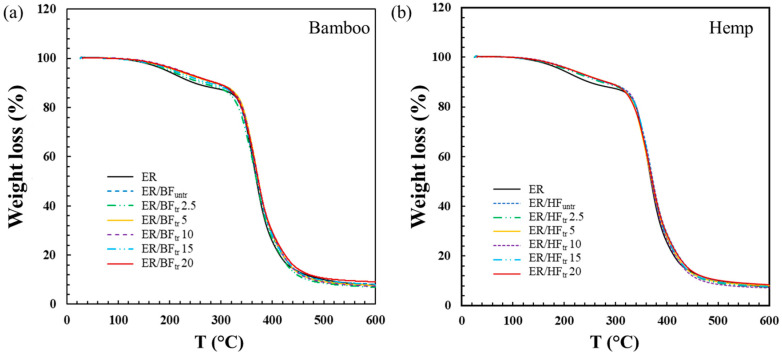
TGA curves of (**a**) BF- and (**b**) HF-reinforced epoxy composites with untreated fibers and fibers treated with Ar + O_2_ gas for different times.

The results of the thermal analysis of the pure epoxy and fiber-reinforced ER composites with untreated fibers and plasma-treated fibers with Ar + O_2_ gas for different times are shown in Figure 5. Weight loss of the epoxy is observed in the first stage at approximately 150 °C due to the released humidity of the composites [62,63]. The ER/BF and ER/HF composites exhibited similar behaviors with the treatment time. In the temperature range of 200–350 °C, the ER blend with fibers exhibited a higher weight loss than the pure ER, which increased with the treatment time. All composites exhibited fiber degradation at 370 °C owing to the degradation of cellulose and hemicellulose composition in the fibers [64]. The phase of lignin was decomposed in the range of 400 to 460 °C with a weight loss percentage of 6%. A higher temperature is needed to decompose hemicellulose, cellulose, and lignin. The remaining component at 500 to 600 °C had an ash content, increasing with the treatment time due to the high degree of crosslinking reaction around the interface of the fibers with ER [65]. The crosslinking improved the interfacial adhesion and mechanical properties of the composites.

### 3.6. Water Contact Angle

**Figure 6:**
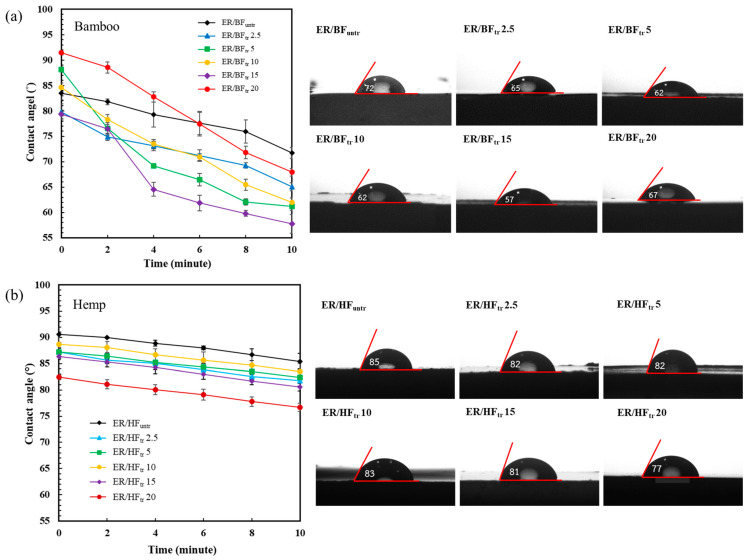
Water droplet contact angles and images of water contact angles at 10 min for the fiber-reinforced epoxy composites with untreated and plasma-treated structures: (**a**) BF and (**b**) HF.

The wettability properties of the composites were calculated using a contact angle measurement, as shown in Figure 6. Water droplets were dropped onto the composite surfaces and automatically recorded from 0 to 10 min. The untreated BF composite (EP/BF_untr_) exhibited a contact angle of 72° after 10 min (Figure 6a), which implies a low surface wettability. The treated samples exhibited decreased water contact angles after 10 min with the increase in treatment time (65, 62, 62, and 57°) due to the high polarity of the plasma treatment, which increased with the 20 min treatment (67°) because of the high crosslinking degree and surface roughness effect of BF_tr_. The ER/HF_untr_ composite exhibited a contact angle of 85° after 10 min (Figure 6b), which decreased with the enhance in the plasma treatment time (82, 82, 83, 81, 77°). The decreasing water droplet contact angle of ER/HF_tr_ indicates a high amount of generated −COOH and −OH groups on the HF_tr_ structure, which reduced the contact angle by its hydrophilicity. However, for BR/BF_tr_ treated for 20 min, the contact angle was increased due to the high crosslinking density between the BF_tr_ and ER matrix. Crosslinking reaction and the enhanced crosslinking amount with time treatment were confirmed by FTIR and TGA results, respectively. Both composite fibers exhibited decreasing contact angles with the increase in the DBD plasma treatment time due to the increases in the surface roughness, porosity, and amount of hydrophilic groups of the fibers subjected to the Ar + O_2_ gas treatment, which led to etching of the surface fibers [48,66]. The long-term plasma treatment affected the water penetration into the fibers and decreased the water resistance of the composite [28,67].

## 4. Conclusions

ER/BF and ER/HF were successfully developed with DBD Ar + O_2_ plasma treatment. The Ar + O_2_ gas plasma treatment significantly enhanced the properties of the bamboo and hemp fiber-reinforced epoxy composites. ER/BFtr was better in terms of tensile strength, while ER/HFtr exhibited a larger improvement in elongation at break. The FTIR spectroscopy confirmed the reaction between epoxy groups of ER with C–O and –NH_2_ groups of the treated fibers, which improved the interfacial adhesion between the fiber surface and ER matrix. The ER/BF_tr_ and ER/HF_tr_ composites exhibited improved flexural strengths and flexural moduli after the Ar + O_2_ plasma treatment due to the improved polarity and roughness of the fiber surface. The SEM analysis showed the fiber surface roughness with a low gap between fibers and ER matrix. The BFs exhibited a gradual increase in surface roughness with the treatment time, while the HFs exhibited a good interfacial adhesion with a low gap. The TGA also confirmed the improved thermal stability and reactions by the plasma treatment with a high weight loss for the long-term Ar + O_2_ plasma treatment. The plasma treatment decreased the water contact angle owing to the increased content of hydrophilic groups, etching, porosity, and roughness of the fiber surface. The ER/BF_tr_ and ER/HF_tr_ composites can be employed in packaging, coating, construction, and electrical applications.

## Figures and Tables

**Table 1:** Notation of the composite material samples (% *w*/*w*).

Sample	Type of Fiber		Epoxy:Hardener (2:1)	Fiber
	Bamboo	Hemp	(%)	(%)
ER/BF_untr_	✓		95	5
ER/BF_tr_ 2.5	✓		95	5
ER/BF_tr_ 5	✓		95	5
ER/BF_tr_ 10	✓		95	5
ER/BF_tr_ 15	✓		95	5
ER/BF_tr_ 20	✓		95	5
ER/HF_untr_		✓	95	5
ER/HF_tr_ 2.5		✓	95	5
ER/HF_tr_ 5		✓	95	5
ER/HF_tr_ 10		✓	95	5
ER/HF_tr_ 15		✓	95	5
ER/HF_tr_ 20		✓	95	5

**Table 2:** Comparison of flexural strengths and flexural moduli of the composites with untreated and plasma-treated fibers.

Sample	Bamboo (B)		Hemp (H)	
	Flexural Stress (MPa)	Flexural Modulus (GPa)	Flexural Stress (MPa)	Flexural Modulus (GPa)
ER/F_untr_	55.9 ± 4.6 ^BC^	7.6 ± 0.6 ^ab^	54.7 ± 0.8 ^I^	7.6 ± 0.1 ^j^
ER/F_tr_ 2.5	54.8 ± 6.8 ^AB^	6.9 ± 0.5 ^bc^	53.9 ± 0.7 ^I^	7.7 ± 0.1 ^i^
ER/F_tr_ 5	50.5 ± 7.8 ^A^	6.6 ± 0.3 ^c^	47.9 ± 0.8 ^J^	6.7 ± 0.1 ^k^
ER/F_tr_ 10	56.8 ± 5.1 ^C^	8.2 ± 0.7 ^a^	56.7 ± 0.9 ^H^	7.8 ± 0.1 ^i^
ER/F_tr_ 15	58.1 ± 5.4 ^C^	8.1 ± 0.7 ^a^	54.4 ± 1.6 ^I^	7.5 ± 0.2 ^j^
ER/F_tr_ 20	57.5 ± 5.9 ^C^	7.7 ± 0.8 ^ab^	62.2 ± 1.1 ^G^	8.6 ± 0.2 ^h^

Ignificantly different mean values of the flexural stress (uppercase letter) and flexural strain (lowercase letter) (*p* < 0.05).

## Data Availability

The data presented in this study are available upon request from the corresponding author.

## Abbreviations and Symbols

ER, epoxy resin; BF, bamboo fibers; HF, hemp fibers; DBD, dielectric barrier discharge; FTIR, Fourier-transform infrared spectroscopy; SEM, scanning electron microscopy; TGA, thermogravimetric analysis.
